# Towards accurate structural characterization of metal centres in protein crystals: the structures of Ni and Cu T_6_ bovine insulin derivatives

**DOI:** 10.1107/S1399004713029040

**Published:** 2013-12-24

**Authors:** Christian Grundahl Frankaer, Susanne Mossin, Kenny Ståhl, Pernille Harris

**Affiliations:** a Department of Chemistry, Technical University of Denmark, Kemitorvet 207, DK-2800 Kgs. Lyngby, Denmark

**Keywords:** bovine insulin, nickel, copper, X-ray absorption spectroscopy, EXAFS, XANES, EPR, photoreduction, radiation damage

## Abstract

Using synchrotron radiation (SR), the crystal structures of T_6_ bovine insulin complexed with Ni^2+^ and Cu^2+^ were solved to 1.50 and 1.45 Å resolution, respectively. The level of detail around the metal centres in these structures was highly limited, and the coordination of water in Cu site II of the copper insulin derivative was deteriorated as a consequence of radiation damage. To provide more detail, X-ray absorption spectroscopy (XAS) was used to improve the information level about metal coordination in each derivative. The nickel derivative contains hexacoordinated Ni^2+^ with trigonal symmetry, whereas the copper derivative contains tetragonally distorted hexacoordinated Cu^2+^ as a result of the Jahn–Teller effect, with a significantly longer coordination distance for one of the three water molecules in the coordination sphere. That the copper centre is of type II was further confirmed by electron paramagnetic resonance (EPR). The coordination distances were refined from EXAFS with standard deviations within 0.01 Å. The insulin derivative containing Cu^2+^ is sensitive towards photoreduction when exposed to SR. During the reduction of Cu^2+^ to Cu^+^, the coordination geometry of copper changes towards lower coordination numbers. Primary damage, *i.e.* photoreduction, was followed directly by XANES as a function of radiation dose, while secondary damage in the form of structural changes around the Cu atoms after exposure to different radiation doses was studied by crystallography using a laboratory diffractometer. Protection against photoreduction and subsequent radiation damage was carried out by solid embedment of Cu insulin in a saccharose matrix. At 100 K the photoreduction was suppressed by ∼15%, and it was suppressed by a further ∼30% on cooling the samples to 20 K.

## Abstract 2

The level of structural detail around the metal sites in Ni^2+^ and Cu^2+^ T_6_ insulin derivatives was significantly improved by using a combination of single-crystal X-ray crystallography and X-ray absorption spectroscopy. Photoreduction and subsequent radiation damage of the Cu^2+^ sites in Cu insulin was followed by XANES spectroscopy.

## 1. Introduction   

Within the field of protein crystallography, an increased understanding of radiation damage from X-rays has developed throughout the last decade (Sliz *et al.*, 2003 ▶; Ravelli & Garman, 2006 ▶; Garman & Nave, 2009 ▶). In particular, metal centres in metalloproteins are sensitive to radiation damage, and for redox-active proteins in which transition metals are actively involved it is crucial that the metal centres are accurately characterized for correct interpretation of the function of the protein.

In X-ray structures, photoreduction of metal centres and the subsequent radiation damage is a well known problem when high-intensity synchrotron radiation (SR) is used. The detailed structure around a metal atom is often distorted owing to radiation damage, which means that looking for loosely bound water molecules in an active site, or determining the detailed coordination geometry of the metal, is not always possible from diffraction experiments, even at high resolution. Complementary techniques to X-ray diffraction (XRD) are thus required to extract information about the metal identity, ligation and redox states. The development of multifunctional beamlines which combine macromolecular crystallography with spectroscopy has facilitated single-crystal spectroscopy experiments on protein crystals with concurrent collection of crystallographic data (Antonyuk & Hough, 2011 ▶; Pearson & Owen, 2009 ▶; De la Mora-Rey & Wilmot, 2007 ▶). Among the spectroscopies used in combination with XRD are X-ray absorption spectroscopy (XAS; Arcovito & della Longa, 2012 ▶; Cotelesage *et al.*, 2012 ▶; Yano & Yachandra, 2008 ▶; Strange *et al.*, 2005 ▶; Hasnain & Strange, 2003 ▶), UV–visible absorption spectroscopy (Hersleth & Andersson, 2011 ▶; Ellis *et al.*, 2008 ▶; Pearson *et al.*, 2004 ▶) and Raman spectroscopy (Katona *et al.*, 2007 ▶; McGeehan *et al.*, 2011 ▶; Hersleth & Andersson, 2011 ▶).

XAS is well suited for studying the redox states and ligation of metals, and includes both extended X-ray absorption fine structure (EXAFS) spectroscopy, which provides detailed information about the radial distribution of atoms, leading to precise determination of bond distances, and X-ray near-edge structure (XANES) spectroscopy, which primarily provides information about oxidation state and connectivity, as the near-edge region is more dominated by multiple scattering events. Compared with the time for a diffraction experiment or the collection of a full EXAFS spectrum, XANES experiments can be performed much more rapidly, which makes this technique advantageous for spectroscopic probing of photoreduction and *in situ* studies of radiation damage. Absorption spectroscopy has previously been used for studying photoreduction in metalloproteins, *e.g.* the putidaredoxin containing an [Fe_2_S_2_] cluster (Corbett *et al.*, 2007 ▶), Fe-containing neuroglobin (Arcovito *et al.*, 2008 ▶), the Mn-complex in photosystem II (Grundmeier & Dau, 2012 ▶), copper nitrite reductase (Hough *et al.*, 2008 ▶) and myoglobin (della Longa *et al.*, 2003 ▶). Direct radiation damage of selenomethione side chains has also been studied by XANES (Holton, 2007 ▶).

In this work, we have studied the structures of Ni^2+^ and Cu^2+^ derivatives of bovine insulin by XRD and XAS, and the coordination geometry of Cu insulin by electron paramagnetic resonance (EPR). Hexameric insulin binds two metal ions by the coordination of three histidine residues (HisB10) to each of the two metal ions. In its natural form the metal is zinc, but insulin is also known to have affinity towards other transition metals, including Mn, Fe, Co, Ni, Cu and Cd (Schlichtkrull, 1956 ▶), all in the +2 oxidation state. In the T_6_ conformation of insulin studied here, both of the metal sites are exposed to the solvent. The coordination sphere for each metal site is thus completed by water molecules, some of which are weakly coordinated and easily exchanged. The T_6_ insulin system thereby provides a well suited model for studying metal centres with labile water molecules. As Cu^2+^ is easily reduced by high-intensity X-ray radiation, the Cu insulin derivative furthermore represents a suitable model system for photoreduction and following radiation damage. Owing to the unique spectral features of Cu^+^ at its X-ray absorption edge, the photoreduction of Cu^2+^ to Cu^+^ can be followed by XANES as a function of the radiation dose delivered to the system. We present suggestions as to how photoreduction can be minimized and follow the radiation-induced structural changes of the Cu sites by in-house XRD.

## 2. Experimental   

### 2.1. Preparation of crystalline nickel and copper insulin   

Single crystals as well as microcrystals of Ni^2+^ and Cu^2+^ insulin were prepared in analogy to the procedures for T_6_ Zn insulin described by Frankaer *et al.* (2012 ▶). Deviations from the reported procedures are reported in the following.

Nickel and copper insulin single crystals were grown using the vapour-diffusion technique. 2 µl of a solution consisting of 7.5 mg ml^−1^ metal-free insulin adjusted to pH 2.0 using aqueous HCl was mixed with 2 µl reservoir solution and equilibrated in a hanging drop against 1 ml reservoir solution with a composition of 0.05 *M* sodium citrate, 15%(*v*/*v*) acetone and 15 m*M* nickel(II) acetate or 7.5 m*M* copper(II) acetate, respectively. In the nickel and copper reservoirs, the pH was adjusted to 7.4 and 7.1, respectively, using aqueous HCl. After 5 d, crystals with dimensions of 200–400 µm were observed. Single crystals were cryoprotected as described by Frankaer *et al.* (2012 ▶) and mounted directly under a 100 K cryostream at the diffractometer before diffraction analysis.

Microcrystal samples of nickel and copper insulin were used for X-ray absorption spectroscopy measurements and were prepared using the method for the preparation of T_6_ Zn insulin microcrystals but with substitution of zinc(II) acetate with nickel(II) acetate or copper(II) acetate.

A powdered sample of Cu insulin microcrystals embedded in a saccharose matrix was obtained using the method described by Ascone *et al.* (2000 ▶). A slurry containing Cu insulin microcrystals and saccharose at a sucrose:insulin ratio of 3:1(*w*:*w*) was prepared by adding 1 ml 75 g l^−1^ sucrose solution to isolated Cu insulin microcrystals crystallized from 25 mg insulin. The slurry was rapidly frozen in 2-propanol/dry ice and lyophilized.

### 2.2. Single-crystal diffraction   

Single-crystal diffraction data for Ni and Cu insulin were collected on beamline I911-2, MAX II at MAXIV Laboratory, Lund, Sweden using a MAR Research MAR165 CCD detector. The photon flux used was estimated to be 1.0 × 10^12^ photons s^−1^ mm^−2^. The scattering properties generally improved after annealing the crystals a few times. The data were processed and scaled using *XDS* and *XSCALE* (Kabsch, 2010 ▶).

In-house single-crystal data from crystals exposed to different radiation doses were collected from Cu insulin crystals using an Agilent Supernova diffractometer. From a large well diffracting crystal, a complete data set to 1.9 Å resolution was collected (CuInsA; Table 1 ▶) while the radiation dose was kept at a minimum (0.01 MGy). High-dose data sets (CuInsB–D; Table 1 ▶) were successively collected on a smaller crystal diffracting to beyond 1.9 Å resolution using a longer exposure time. All in-house diffraction data were collected at 100 K using Cu *K*α radiation (λ = 1.5419 Å) with a photon flux of 1.0 × 10^10^ photons s^−1^ mm^−2^ at the sample. The crystal sizes were carefully determined from photographs using the *CrysAlis^Pro^* software (Agilent Technologies) and radiation doses were calculated by *RADDOSE* (Murray *et al.*, 2004 ▶; Paithankar *et al.*, 2009 ▶) and are listed in Table 1 ▶. Data were processed and scaled using the *CrysAlis^Pro^* software (Agilent Technologies).

Using the peptide chain from the T_6_ Zn insulin structure (PDB entry 4e7t; Frankarr *et al.*, 2012 ▶) as a starting model, the structures were refined using *REFMAC*5 (Murshudov *et al.*, 2011 ▶) and *PHENIX* (Adams *et al.*, 2010 ▶). Model building and editing were carried out using *WinCoot* (Emsley *et al.*, 2010 ▶). The structures were validated using *PROCHECK* (Laskowski *et al.*, 1993 ▶), *WHAT_CHECK* (Hooft *et al.*, 1996 ▶) and the structure-analysis server *STAN* (Kleywegt & Jones, 1996 ▶). Data-collection and refinement statistics for all crystals are summarized in Table 1 ▶.

#### 2.2.1. Ni insulin synchrotron structure   

Two Ni atoms were included, and the side chains of residues GlnB4.1, ValB12.1, LeuB17.1, CysA11.2 and GlnB4.2 were modelled in two alternate conformations. A total of 80 water molecules were inserted. Restrained refinement was carried out in *PHENIX* and H atoms were included. The atomic displacement factors for the peptide chain were refined by a combination of TLS refinement and isotropic refinement. The TLS domains were as follows: residues 1–8 and 13–19 in the A chains, residues 9–18 in the B chains and a group containing residues 23–27 of two adjacent B chains. Other atoms were refined isotropically. Validation showed that only one residue, SerA9.1, was in the outlier region of the Ramachandran plot (as defined by Kleywegt & Jones, 1996 ▶).

#### 2.2.2. Cu insulin synchrotron structure   

Two Cu atoms were inserted, and the side chains of residues GlnB4.1, ValB12.1, LeuB17.1, CysA11.2 and ValB12.2 were modelled in two alternate conformations. 98 water molecules were inserted in total and H atoms were included. The refinement procedure was analogous to that used for Ni insulin. Validation showed that only one residue, SerA9.1, was in the outlier region of the Ramachandran plot.

#### 2.2.3. Cu insulin in-house structures   

The evolution of radiation damage was studied from comparison of four models corresponding to different values of absorbed dose from 0.01 to 0.30 MGy. It should be emphasized that the three data sets (CuInsB–D) were collected from the same crystal, which explains the similar unit-cell parameters and mosaicity values observed for these structures. In the subsequent structure refinement two Cu atoms were included in each model. The C-­terminal residue AlaB30.1 was disordered in all four structures and was therefore not included. PheB1.1 was not modelled in CuInsA and CulnsD. The side chains of residues ValB12.1 and CysA11.2 were modelled in two alternate conformations for all structures, and for CuInsB, CuInsC and CuInsD further alternate conformations were found for the side chains of residues GluB13.1 and ValB12.2. A number of water molecules ranging from 52 to 68 was included in each of the structures (see Table 1 ▶), and all structures were refined to a resolution of 1.9 Å, resulting in *R* factors below 0.17 and *R*_free_ factors below 0.24. The residue SerA9.1 was in the outlier region in the Ramachandran plot for all four structures. The outlier region also included SerA9.2 in CuInsB–D and ProB28.1 in CuInsA.

### 2.3. X-ray absorption spectroscopy   

Ni and Cu *K*-edge X-ray absorption spectra were recorded on beamline I811 at the synchrotron at MAXIV Laboratory, Lund, Sweden (Carlson *et al.*, 2006 ▶) using a Si(111) double-crystal monochromator detuned 60% at 9333 and 9829 eV for Ni and Cu insulin, respectively. The samples were mounted in 1 mm thick sample holders (Frankaer *et al.*, 2011 ▶) and were cooled to either 20 or 100 K in a cryostat using liquid helium or liquid nitrogen, respectively. Fluorescence data were collected using a PIPS PD-5000 (passivated implanted planar silicon) detector from Canberra with the scan ranges and times listed in Table 2 ▶. In order to ensure that no radiation damage of the sample had taken place, or at least that it was minimized, a fast scan was performed after the collection of each EXAFS spectrum. For Cu insulin the sample was renewed between each EXAFS scan.

The following reduction and analysis of EXAFS data were carried out using *WinXAS* (Ressler, 1998 ▶) and *EXCURVE* (Gurman *et al.*, 1984 ▶, 1986 ▶; Binsted *et al.*, 1991 ▶) in accordance with the procedure described by Frankaer *et al.* (2012 ▶). Calculation of XANES spectra by finite-difference methods (FDM) was performed using *FDMNES* (Joly, 2001 ▶).

The photoreduction of Cu insulin at different temperatures with and without saccharose protection was monitored by XANES. The XANES data-collection specifications and dose calculations as performed by *RADDOSE* (Murray *et al.*, 2004 ▶; Paithankar *et al.*, 2009 ▶) are summarized in Table 2 ▶. The XANES spectra were energy-calibrated using an internal Cu foil reference sample and were background-subtracted and normalized using *ATHENA* (Newville, 2001 ▶; Ravel & Newville, 2005 ▶). The signal from copper in oxidation state +1 was extracted from the peak located at 8983 eV (Kau *et al.*, 1987 ▶). This peak was isolated by the subtraction of a fast-scan XANES spectrum collected at 20 K, in which no peak was observed at 8983 eV and which hence was obtained before photoreduction takes place. The area under the isolated peak was calculated by fitting a Gaussian function to the left-hand side of the peak, since the right-hand side is difficult to resolve owing to its location very close to the absorption edge.

### 2.4. Electron paramagnetic resonance   

EPR was recorded on solid microcrystalline Cu insulin. The sample was recorded at room temperature (RT) and at 77 K using a liquid-nitrogen finger dewar in the ST4102 resonator of an X-band Bruker EMX EPR spectrometer. The microwave frequency was 9.34 GHz, the microwave power was 5 mW, the modulation frequency was 100 kHz and the modulation amplitude was 8 G. The spectrum was recorded over three sweeps. The spectrum of the empty tube was subtracted and the spectrum was fitted using the spin Hamiltonian-based program *W*95*EPR* (Neese *et al.*, 1996 ▶).

## 3. Results   

### 3.1. Nickel insulin   

**Figure 1:**
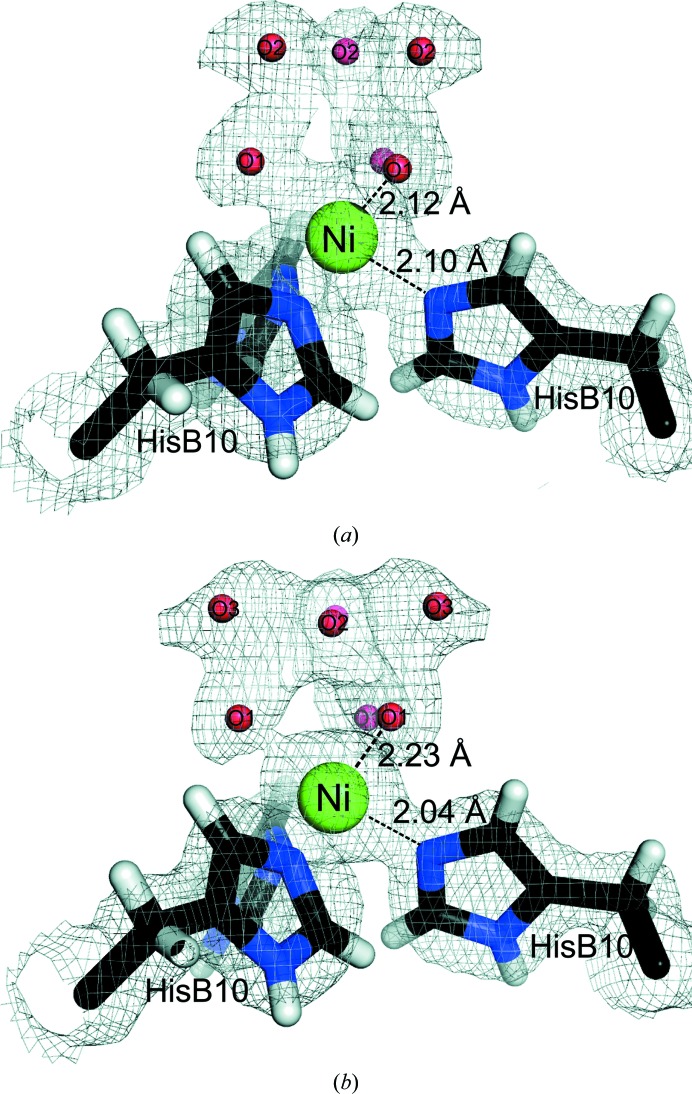
(*a*) Nickel site I and (*b*) nickel site II in the 1.50 Å resolution Ni insulin structure. Ni^2+^ is hexacoordinated by three HisB10 residues and three water molecules (red) coordinating to Ni in both sites. Distances to the first coordination sphere as determined by single-crystal X-ray diffraction are shown. The *σ*_A_-weighted 2*F*_o_ − *F*_c_ maps are contoured at 1.0σ.

In the crystal structure of insulin co-crystallized with nickel, the hexamers were found to adopt the T_6_ conformation in analogy to the T_6_ zinc insulin structure (Frankaer *et al.*, 2012 ▶). The structure contains two Ni^2+^ ions exposed to the solvent in both of the open T_3_ sites, as shown in Fig. 1 ▶. The Ni atoms are hexacoordinated, with the coordination sphere consisting of three equivalent imidazole N atoms and O atoms from three equivalent water molecules owing to the threefold symmetry. The distances between Ni and the O atoms of the water molecules are 2.12 and 2.23 Å in sites I and II, respectively.

**Figure 2:**
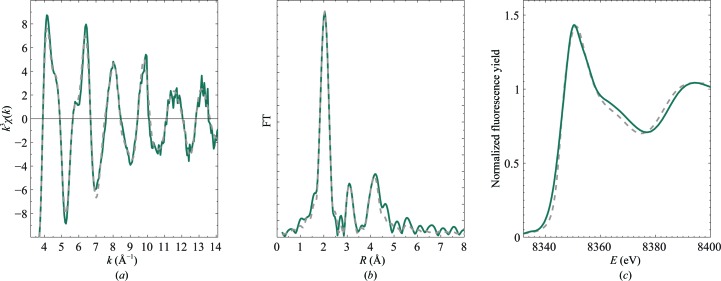
(*a*) *k* ^3^-weighted EXAFS and (*b*) radial distribution function calculated as the modulus of the phase-corrected Fourier transform of T_6_ nickel insulin. Experimental spectra are shown in blue and simulated spectra in grey (dashed lines) using the parameters from the restrained refinement given in Table 3 ▶. (*c*) Ni insulin XANES. The experimental XANES (blue) is compared with XANES calculated from a 4.5 Å cluster around the Ni atom using the refined coordinates from EXAFS (dashed line).

The extracted *k* ^3^-weighted EXAFS spectrum and the modulus of the phase-corrected Fourier transform of Ni insulin are presented in Fig. 2 ▶. The shape of the *k* ^3^-weighted χ(*k*) for Ni insulin has a high resemblance to that of T_6_ Zn insulin reported by Frankaer *et al.* (2012 ▶), indicating an analogous pseudo-octahedral coordination. Distances and Debye–Waller factors, as optimized from a restrained EXAFS refinement using coordinates from the SR structure, are presented in Table 3 ▶ and the fit is shown in Figs. 2 ▶(*a*) and 2 ▶(*b*).

A XANES spectrum calculated by the FDM method is shown in Fig. 2 ▶(*c*) using the coordinates from the model optimized by EXAFS. There is good agreement between the experimental and calculated spectra. The high-intensity white line is in agreement with the XANES spectra reported for other hexacoordinated nickel complexes (Colpas *et al.*, 1991 ▶), thereby verifying the pseudo-octahedral coordination.

### 3.2. Copper insulin   

**Figure 3:**
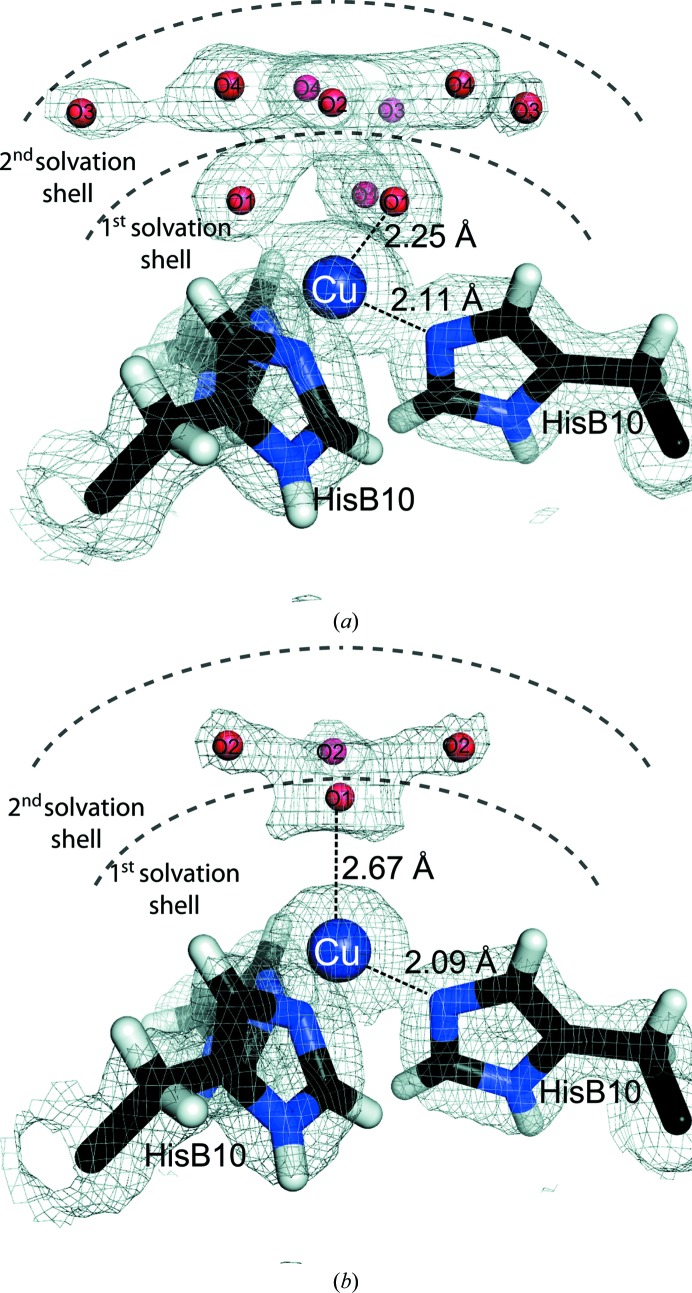
(*a*) Copper site I and (*b*) copper site II in the 1.45 Å resolution Cu insulin structure. Cu^2+^ is coordinated to three HisB10 residues and three water molecules (red) in site I, whereas the coordination is more ambiguous in site II. Distances to the first coordination sphere as determined by single-crystal X-ray diffraction are shown. The *σ*_A_-weighted 2*F*_o_ − *F*_c_ maps are contoured at 1.0σ.

Hexameric copper insulin is found to adopt the T_6_ conformation in all of the crystal structures reported here. The synchrotron crystal structure of copper insulin contains two Cu ions coordinated by the HisB10 residues in both T_3_ sites, as shown in Fig. 3 ▶. In site I a hexacoordinated copper is observed with a Cu–water distance of 2.25 Å. In site II the coordination has a more tetrahedral character, in which one water molecule can be modelled in the first solvation shell on the threefold symmetry axis at 2.67 Å from the Cu atom, as shown in Fig. 3 ▶. However, the electron density is still reminiscent of a pseudo-octahedral coordination. Also, a weaker electron density is observed in the second solvation shell, which indicates deter­ioration of the water structure as a consequence of radiation damage.

#### 3.2.1. Radiation damage monitored by XANES and in-house XRD   

**Figure 4:**
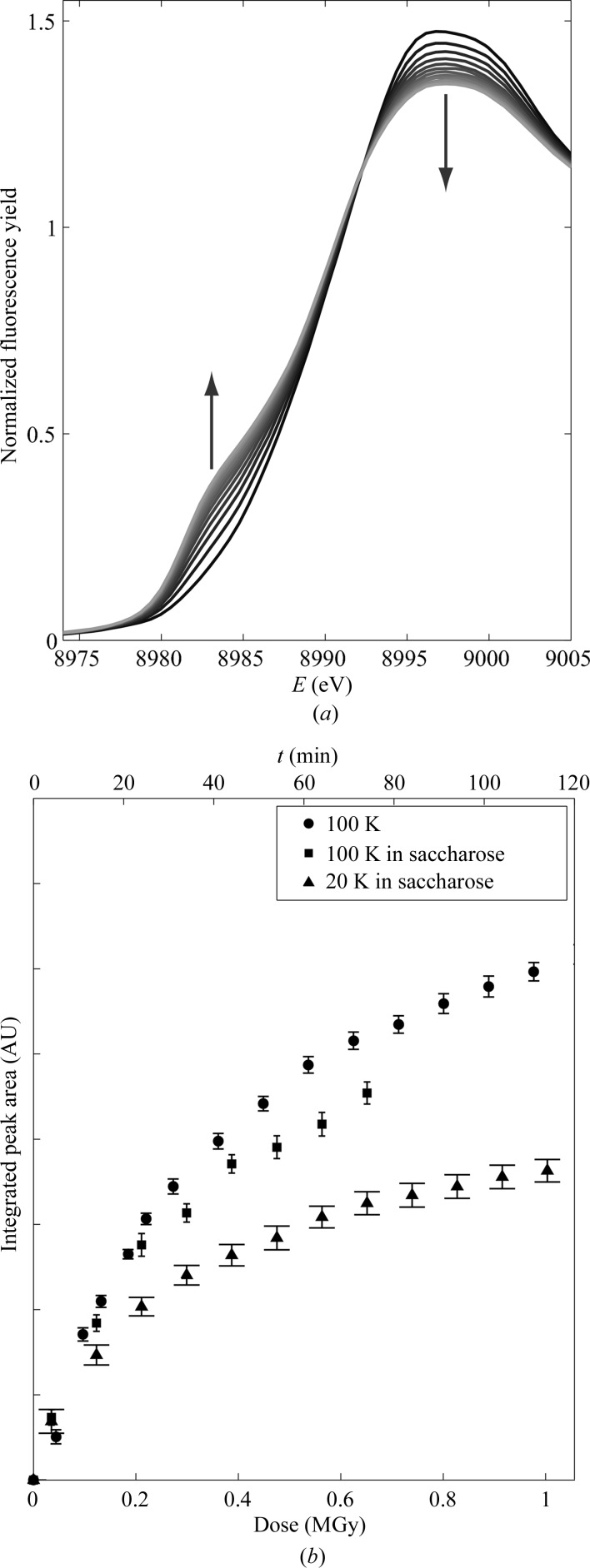
(*a*) Variation of the XANES spectrum of Cu insulin collected at 100 K as a function of exposure time/dose. The peak appearing at 8983 eV originates from the formation of Cu^+^. Measurements were performed with 10 min between each measurement, starting at *t* = 10 min (black) and ending at *t* = 110 min (light grey). Arrows show the spectral evolvement as photoreduction propagates. (*b*) The integrated peak area of the peak at 8983 eV, which is proportional to the amount of Cu^+^ as a function of exposure time/dose for Cu insulin samples prepared with and without saccharose at 100 K and with saccharose at 20 K. The amount of Cu^+^ is shown as the integrated area under the peak occurring at 8983 eV. Error bars indicate the uncertainty of the integration.

XANES spectra collected on Cu insulin at 100 K as a function of radiation dose (ranging from approximately 0.1 to 1.0 MGy) are shown in Fig. 4 ▶(*a*). As the radiation dose increases a peak arises at 8983 eV. This peak originates from the 1*s*→4*p* electronic transition of copper in oxidation state +1 (Kau *et al.*, 1987 ▶), thereby showing that photoreduction of Cu^2+^ to Cu^+^ takes place. Furthermore, it is seen in Fig. 4 ▶(*a*) that the intensity of the white line decreases as photoreduction takes place. This indicates a change in the coordination surroundings towards tetrahedral geometry (Kau *et al.*, 1987 ▶).

**Figure 5:**
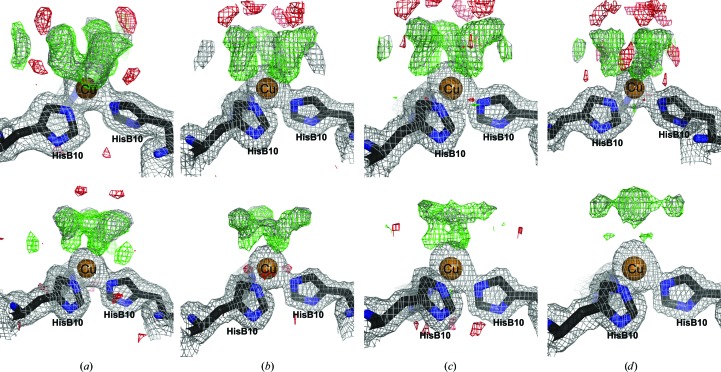
Difference electron-density maps of the water structure in copper site I (top panel) and copper site II (bottom panel) after different radiation doses: (*a*) 0.00–0.01 MGy, (*b*) 0.01–0.06 MGy, (*c*) 0.06–0.12 MGy, (*d*) 0.24–0.30 MGy. The *σ*_A_-weighted 2*F*_o_ − *F*_c_ maps (grey) are contoured at 1.0σ. The difference *F*_o_ − *F*_c_ maps (green/red) are contoured at ±3.0σ, respectively.

In the crystal structures solved from data collected using our in-house equipment, in which the crystals were exposed to different radiation doses, the copper sites are shown in Fig. 5 ▶. The water molecules have been removed from the structures and difference maps have been calculated. At low radiation doses the difference maps indicate hexacoordinated copper in both copper sites, which is in agreement with the XANES results and analogous to the Ni insulin structure. At higher doses no significant changes in the coordination of the Cu I site are observed, whereas the Cu II site is seen to change with increasing radiation dose. This rearrangement is in agreement with the decrease in white-line intensity seen in the XANES spectra (Fig. 4 ▶ *a*).

Generally, the water structure around the Cu sites was difficult to model. The water–water distances appear to be closer than normal hydrogen-bond distances, down to 2.0 Å. This problem could not be solved by decreasing the occupancy or increasing the lower cutoff for intermolecular water–water distances. In our structures no significant difference in the first water shell around the Cu I site was observed in the four different structures. In the second water shell, the water molecule next to the coordinating water apparently moves away from the Cu site with increasing radiation dose. The water–water distance also increases, from 2.00 to 2.35 Å, which indicates minor changes, in site I. However, these changes were only monitored and were not explained by the final models.

#### 3.2.2. Minimizing photoreduction   

The evolution of the photoreduction in Cu insulin samples prepared without saccharose at 100 K, with saccharose at 100 K and with saccharose at 20 K is shown in Fig. 4 ▶(*b*). Cu^+^ formation is probed by the peak appearing at 8983 eV and the relative amount of Cu^+^ has been calculated by integration of this peak, as described in §[Sec sec2.3]2.3. The peak areas are plotted as a function of radiation dose/exposure time. As seen from this figure, the amount of Cu in oxidation state +1 increases with increasing radiation dose. Comparing the three samples, it is seen that photoreduction is slowed by approximately 15% by embedding the protein in a saccharose matrix and by a further 30% by cooling the saccharose-protected sample from 100 to 20 K.

#### 3.2.3. Coordination of Cu in an undamaged sample   

**Figure 6:**
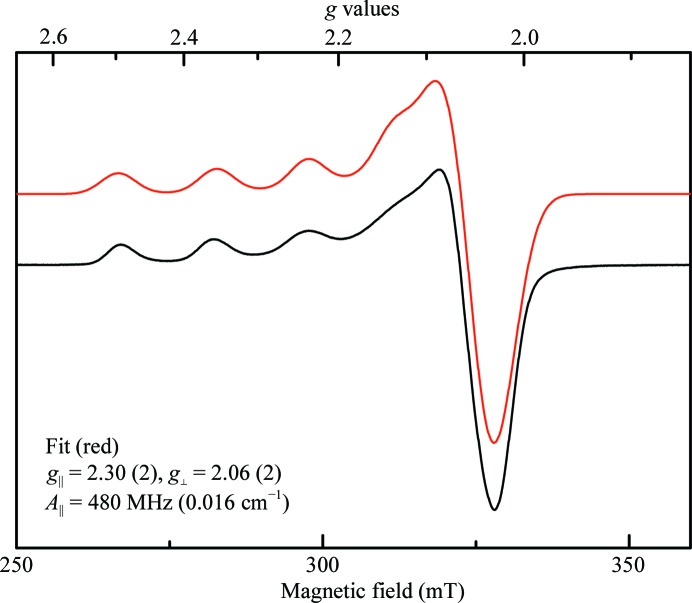
Experimental (black) and fitted (red) EPR spectra of microcrystalline Cu^2+^ insulin measured at RT. The spectral parameters *g*_⊥_, *g*_∥_ and *A*_∥_ are determined by the fit and are printed in the figure.

The experimental EPR spectrum of microcrystalline Cu insulin is shown in Fig. 6 ▶ (black line). The experimental spectrum has been fitted (red line) with the usual axial spin Hamiltonian model used to model EPR spectra of Cu^2+^ (Neese *et al.*, 1996 ▶), with *g*-values *g*_∥_ = 2.30 (2), *g*_⊥_ = 2.06 (2) and with the parallel component of the coupling constant to the nuclear spin of copper being *A*_∥_ = 480 (30) MHz [0.016 (1) cm^−1^]. The perpendicular component *A*_⊥_ was not resolved and therefore was not well determined, but was set to 30 MHz in the fitted spectrum shown. The line shape was Gaussian. The differences between the two copper sites and the coupling to the nitrogen nuclei were not resolved. The parameters correspond to a type II Cu^2+^ protein with two nitrogen donors and two oxygen donors (Peisach & Blumberg, 1974 ▶) and a predominantly tetragonal site geometry (Savelieff *et al.*, 2008 ▶). This corresponds to Cu^2+^ being coordinated to two imidazole N atoms from histidine residues and to two water molecules in the plane of the 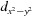 orbital, with the final imidazole on the *z* axis.

For Cu^2+^ with six nitrogen or oxygen donors the trigonal symmetry will give rise to a degenerate ground state of Cu^2+^ owing to the *d* ^9^ electronic structure. This will be Jahn–Teller unstable and therefore some geometric distortion is expected to take place. Nevertheless, in all crystal structures copper is bound in positions of *C*_3_ symmetry and coordinates to three equivalent histidine residues just as in the Ni^2+^ and Zn^2+^ analogues. EPR clearly demonstrates that in the low-dose crystal the actual ligand field experienced by the copper centre in both sites I and II is close to tetragonal and therefore one histidine residue must be different from the other two. In order to investigate the discrepancy between the crystallographic *C*_3_ site symmetry and the EPR data, we proceeded to compare different geometry models with the XAS data.

**Figure 7:**
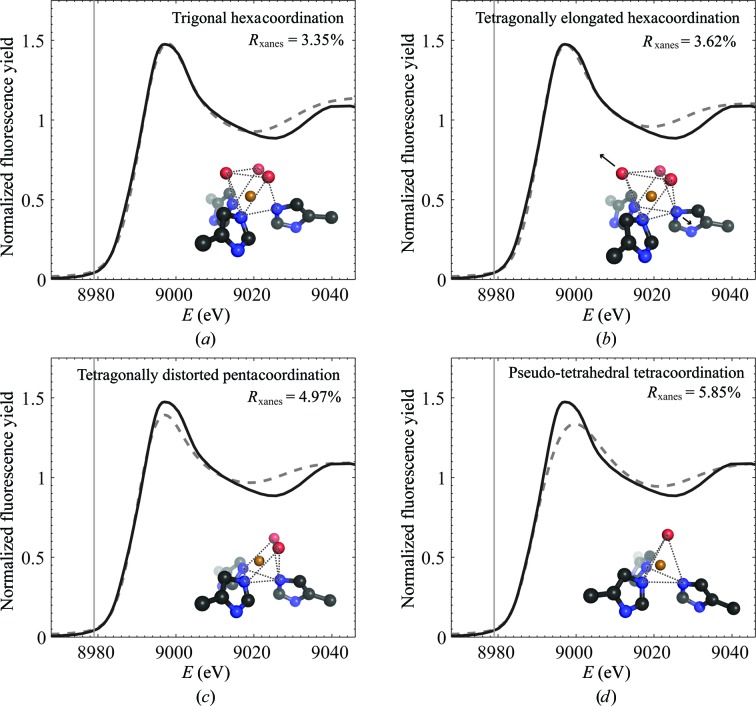
Comparison of the experimental Cu *K*-edge XANES spectrum of saccharose-protected Cu insulin collected at 20 K within 10 min (continuous line) with theoretical XANES spectra (dashed) calculated from a 4.5 Å cluster around the Cu atom using coordinates optimized from EXAFS refinement for (*a*) a trigonal hexacoordinated model (*C*_3_), (*b*) a tetragonally elongated hexacoordinated model, (*c*) a tetragonal pentacoordinated model and (*d*) a pseudo-tetrahedral model (*C*_3_). The *K* edge for metallic copper is shown at 8979 eV.

For XAS analysis, four different copper geometries were tested by building models with trigonal symmetric hexacoordination, tetragonally elongated hexacoordination, tetragonally distorted square-pyramidal geometry (pentacoordination) and trigonal symmetric pseudo-tetrahedral geometry (tetracoordination). Coordinates were taken from the SR crystal structure and the presence and position of the O atoms from water were modified according to each of the four geometries. Before calculation of XANES spectra each model was optimized by a constrained EXAFS refinement in the *k* range 2.8–13.3 Å^–1^. The structural parameters of the optimized geometries are presented in Table 4 ▶. XANES spectra calculated from models as optimized by EXAFS are presented in Fig. 7 ▶.

**Figure 8:**
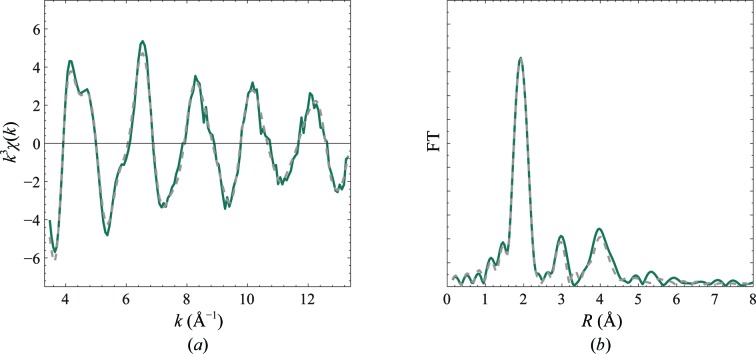
(*a*) *k* ^3^-weighted EXAFS and (*b*) radial distribution function calculated as the modulus of the phase-corrected Fourier transform of T_6_ copper insulin. Experimental spectra are shown in blue and simulated spectra are shown in grey (dashed lines) from a tetragonally distorted hexacoordinated Cu using the parameters from the constrained refinement given in Table 5 ▶.

As seen in the calculated XANES spectra in Fig. 7 ▶, the intensity of the white line decreases with decreasing coordination number. The high-intensity white line observed in the experimental spectrum thus shows that copper is hexacoordinated. The poor fit to the pseudo-tetrahedral model further confirms the presence of type II copper sites. As seen in Table 4 ▶, it is noteworthy that the trigonal hexacoordinated model fits the EXAFS data poorly, which is in agreement with the EPR results and with the general coordination preferences of Cu. Hence, the best fits from both the EXAFS refinement as well as the calculated XANES are obtained from the tetragonally elongated hexacoordinated model: *R*_exafs_ = 14.38% and *R*_xanes_ = 3.62%. The extracted *k* ^3^-weighted EXAFS spectrum and the modulus of the phase-corrected Fourier transform of Cu insulin are presented in Fig. 8 ▶, to­gether with the fit for the tetragonally elongated hexacoordinated model. The optimized distances and Debye–Waller factors of this model are presented in Table 5 ▶. It is seen that all three distances from copper to nitrogen ligands in the histidine residues are very similar and that the tetragonal distortion is thereby most prominent for the axial water molecule, which is a further 0.2 Å away from Cu^2+^.

## 4. Discussion   

### 4.1. General conformation   

Hexameric insulin was successfully crystallized with di­valent cations of nickel and copper, and the structures were solved. The T_6_ conformation was observed in all structures and the structures were compared with the zinc T_6_ structure and other structures in the PDB by superposing independent T_2_ dimers in *SUPERPOSE* (Krissinel & Henrick, 2004 ▶), in which the C^α^ displacements were minimized and the root-mean-square deviations (r.m.s.d.s) were calculated. Both structures show high resemblance to the bovine zinc T_6_ insulin structures deposited in the PDB [PDB entries 2a3g (Smith *et al.*, 2005 ▶) and 4e7t (Frankaer *et al.*, 2012 ▶)], with an r.m.s.d. below 0.3 Å. In analogy to bovine zinc T_6_ insulin, comparison with the structures of human Ni insulin and Cu insulin [PDB entries 3exx (Prugovečki *et al.*, 2009 ▶) and 3tt8 (Prugovečki & Matković-Čalogović, 2011 ▶)] show larger discrepancies (r.m.s.d.s around 1.3 Å) owing to a different conformation of the B1.2–B3.2 chain. The weak determination of the B1.2–B3.2 residues may be a consequence of a partially disordered N-terminus of the B chain.

### 4.2. Coordination of nickel in Ni insulin   

The octahedral coordination of nickel in insulin observed in the crystal structure is in good agreement with the results obtained by XAS. In general, the Ni–O^w1^ distances are slightly longer in the crystal structure (2.18 Å on average) compared with the distance as refined by EXAFS (2.10 Å). Similar deviations were observed between the crystal structure and the EXAFS results for T_6_ Zn insulin (Frankaer *et al.*, 2012 ▶), which was explained by the higher radiation doses in the diffraction experiment. The observed nickel coordination is generally in very good agreement with the human Ni insulin structure (PDB entry 3exx; Prugovečki *et al.*, 2009 ▶).

### 4.3. Photoreduction of copper in Cu insulin   

The sensitivity to photoreduction is first and foremost dependent on the metal coordinated to the protein. Whereas Ni^2+^ is very stable, Cu^2+^ can easily be reduced to Cu^+^ in the X-­ray beam. The problem with photoreduction of copper centres is well known and preservation by lyophilizing protein solutions in saccharose has previously been reported for haemocyanin and haemoglobin (Ascone *et al.*, 2000 ▶). By keeping the protein in a solid phase, the mobility of damaging species is reduced because free diffusion is hindered.

As demonstrated in Fig. 4 ▶(*b*), photoreduction is suppressed when the Cu insulin crystals are embedded in a saccharose matrix and is even further reduced on cooling to 20 K (by approximately 40% in total). The experiments showed that this preservation technique also can be performed on microcrystalline samples, which makes this method even more versatile.

The characteristic feature in the XANES spectrum from Cu in oxidation state +1 makes it possible to suggest a mechanism by which the photodegradation proceeds as a function of radiation dose ϕ. As seen from the successive collected XANES spectra, photoreduction takes place immediately after exposure. Compared with the evolution of the electron density in the in-house crystal structures (Fig. 5 ▶), the structural changes around copper in site II seem to be detectable at radiation doses of around 0.1 MGy and above. Thereby, the radiation damage to the water coordination could be initiated by the photoreduction of Cu^2+^ to Cu^+^.

**Figure 9:**
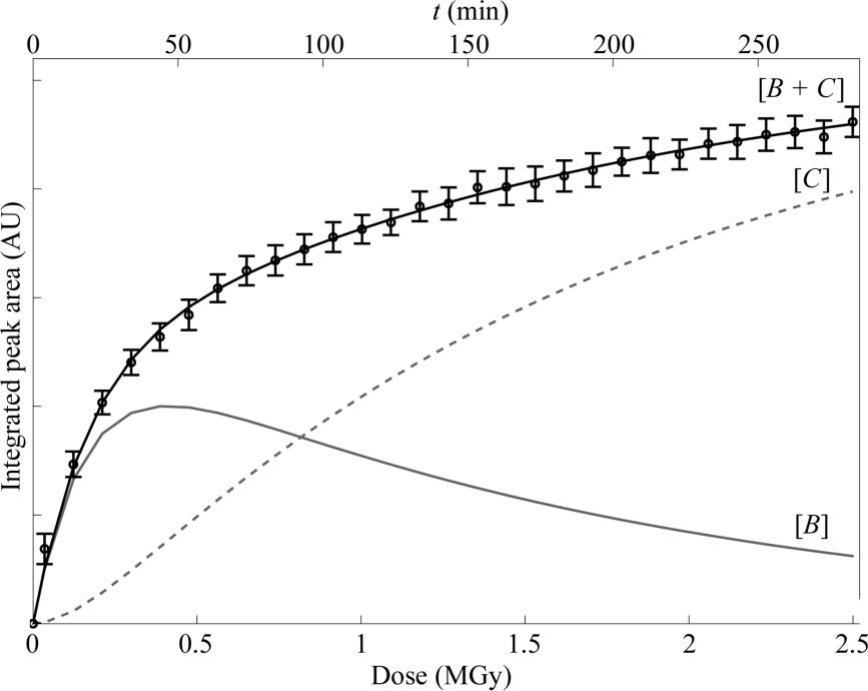
Integrated area under the peak at 8983 eV, which is proportional to the amount of Cu^+^, as a function of exposure time for a Cu insulin sample prepared with saccharose at 20 K. The formation of Cu^+^ has been modelled using a pre-equilibrium mechanism which includes two different Cu^+^ species, *B* and *C*, with concentrations [*B*] (solid grey line) and [*C*] (dashed grey line), respectively (see equation 1). The total amount of Cu^+^ from the model [*B* + *C*] (black solid line) has been fitted to the experimental data (circles).

**Figure 10:**
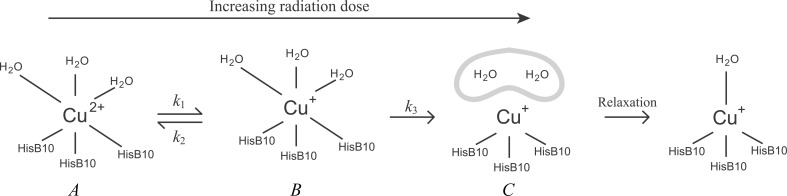
Suggested mechanism for the photoreduction of Cu insulin. The redox reaction occurs as a pre-equilibrium between the type II copper insulin species, which is followed by an irreversible step in which the water structure of the reduced copper deteriorates (indicated by a grey cloud of water) and then stabilizes. For simplicity, the different species are renamed *A*, *B* and *C*.

The photoreduction was monitored for approximately 300 min for a Cu insulin sample embedded in saccharose at 20 K and the formation of Cu^+^ over the entire series is shown in Fig. 9 ▶ (circles). As seen from the figure, the reaction does not seem to reach equilibrium within the first 5 h. Instead, Cu^+^ builds up at an approximately constant rate after approximately 1 MGy. A similar trend has been observed for the reduction of Fe^3+^ in putidaredoxin after long exposures (doses of up to 12 MGy) by Corbett *et al.* (2007 ▶). Although exponential curves seem to accurately reproduce the data in the low-dose range (<1 MGy), it is noteworthy that the rate at which the reduced species build up seems to be linear after doses exceeding 1 MGy. This suggests a pre-equilibrium mechanism (Rae & Berberan-Santos, 2004 ▶) following the scheme shown in Fig. 10 ▶.

In Fig. 10 ▶ a reversible redox reaction between the hexacoordinated tetragonally distorted copper insulin species *A* and *B* is followed by a step in which the water structure is deteriorated: species *C*. Following this reaction as a function of radiation dose ϕ, rate constants *k*_1_, *k*_2_ and *k*_3_ were determined from a numerical solution of the differential equation system
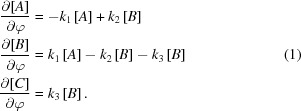
This resulted in *k*_1_ = 5.9 MGy^−1^, *k*_2_ = 0.3 MGy ^−1^ and *k*_3_ = 1.2 MGy^−1^. The concentrations of *B* and *C*, and the total amount of copper in oxidation state +1, [*B* + *C*], are shown individually in Fig. 9 ▶. The high value of *k*_1_ compared with *k*_2_ shows that the equilibrium between Cu^2+^ and Cu^+^ is shifted to the right. The rearrangement of the water structure in *B* and the subsequent relaxation is slower and acts as a way to stabilize the Cu^+^ centres, thereby dragging the pre-equilibrium to the right.

### 4.4. Coordination of copper   

The EPR results presented here are in excellent agreement with previous Cu insulin EPR results by Brill & Venable (1968 ▶), who found that the two Cu sites had identical geometry. Similar type II Cu^2+^ complexes involving histidine ligands have been observed in recent EPR studies of Cu^2+^-containing amyloid-β (Shin & Saxena, 2008 ▶; Jun *et al.*, 2009 ▶). Comparison with other biological copper(II) complexes, such as Cu–salicylate complexes (Valko *et al.*, 1990 ▶), shows good agreement with tetragonal geometry with two N and two O atoms in the equatorial plane.

As previously shown, Cu insulin is very sensitive to photoreduction and subsequent radiation damage of the Cu centres. Hence, the coordination of copper as determined by the different X-­ray techniques depends on the radiation dose. A tetragonally distorted hexacoordinated geometry of copper in both sites (species *A* in Fig. 10 ▶) is in agreement with the results from the non-destructive EPR experiment, the low-dose X-ray experiments (in-house XRD) and the low-temperature XAS (saccharose-protected samples at 20 K). The distances from Cu to the N atoms of histidine residues are generally longer in crystal structures (2.10 Å on average) compared with the distances as refined by EXAFS (1.98/2.02 Å) and, in analogy to the nickel structure, a similar trend is observed for the copper–water distances. In general, the Cu-ligation distances determined in this study fall well within the range of both Cu–N and Cu–­water distances observed in other protein structures containing copper type II centres (Abriata, 2012 ▶), and the hexacoordinated coppers observed in the low-dose structures are in good agreement with the coordination of copper in the human Cu insulin structure (PDB entry 3tt8; Prugovečki & Matković-Čalogović, 2011 ▶).

Whereas an accurate characterization of the copper ligation is excellently provided by XAS and EPR in combination with XRD, the information about water coordination in the second solvation shell is limited using these techniques. For XAS and EPR the limiting factor is the spectral resolution, whereas for XRD the limit is determined by the degree of radiation damage, which induces structural changes around the metal atoms. As illustrated by the crystal structures presented here, a chemically reasonable model is difficult to obtain as the intermolecular water distances are unrealistically low. This seems to be a general problem. Comparison with other high-resolution insulin structures such as the 1.0 Å resolution structure of human T_6_ insulin with zinc (PBD entry 1mso; Smith *et al.*, 2003 ▶) as well as the 1.12 Å resolution structure of human Cu insulin (PDB entry 3tt8; Prugovečki & Matković-Čalogović, 2011 ▶) reveals similar unrealistically short water–water distances close to the metal sites.

In theory, the tetragonally distorted copper coordination of Cu^2+^ is in conflict with the *C*_3_ symmetry observed in the Cu insulin crystal structures, as it will cause the distance from Cu to one of the three histidines as well as to one of the water molecules to differ from the other two. The elongation is more expressed in the Cu–water bond since the water molecules are less restricted. Nevertheless, if the tetragonal elongation is equally distributed among, or resonating between, the three symmetry-related N—Cu—O axes, the average effect would not break down the crystallographic *C*_3_ symmetry of the metal site.

## 5. Conclusions   

The coordination of Cu and Ni in bovine insulin derivatives was studied by combining XAS with crystallography. Both nickel and copper were found to be hexacoordinated and the distances between the metal and its ligands were very precisely determined using EXAFS. Furthermore, EPR measurements of the Cu^2+^ insulin derivative clearly revealed the presence of type II copper sites in which copper adopts a tetragonal coordination. We have demonstrated that crystallography must be complemented by other techniques if structural details are to be resolved around the metal sites, in particular for labile ligated sites such as the water molecules present in the insulin T_6_ conformation studied here. To some extent the sensitivity towards radiation damage from the X-­radiation depends on the actual ligation of a metal, but it primarily depends on the type of metal and its oxidation state. Nickel insulin containing octahedrally coordinated Ni^2+^ was found to be stable throughout the X-ray experiments, whereas the Cu^2+^ in copper insulin suffered from photoreduction in which Cu^+^ was formed and the coordination sphere was disrupted.

The importance of efficient protection against radiation damage was illustrated by following the photoreduction (primary damage) by XANES as a function of radiation dose and by monitoring the structural changes (secondary damage) around copper at different radiation doses by crystallography using an in-house X-ray source. At 100 K, disruption of the water structure in Cu site II was detected at doses above 0.1 MGy. We found that the photoreduction could be supressed by approximately 15% by embedding the protein in a saccharose matrix and by a further 30% by cooling the saccharose-protected sample to 20 K. This study thus recommends the use of the solid saccharose matrix-embedment protocol and liquid helium-based cooling for studying photoreduction-active metals in biological systems.

## Supplementary Material

PDB reference: Cu insulin, 4m4f

PDB reference: 4m4h

PDB reference: 4m4i

PDB reference: 4m4j

PDB reference: 4m4l

PDB reference: Ni insulin, 4m4m

## Figures and Tables

**Table 1:** XRD data-collection and refinement statistics Values in parentheses are for the outermost resolution shell.

	Synchrotron†		In-house			
	Ni insulin	Cu insulin	CuInsA	CuInsB	CuInsC	CuInsD
PDB entry	4m4m	4m4l	4m4f	4m4h	4m4i	4m4j
Experimental parameters						
Crystal volume (mm^3^)	0.0080	0.0080	0.0125	0.0006	0.0006	0.0006
Wavelength ()	1.040	1.040	1.5419	1.5419	1.5419	1.5419
Exposure time (s)	01070	01800	0996	05660	566011320	2464028300
Radiation dose (MGy)	0.000.50	0.000.85	0.000.01	0.00.06	0.060.12	0.240.30
Temperature (K)	100	100	100	100	100	100
Data-collection statistics						
Resolution ()	16.781.50 (1.541.50)	16.871.45 (1.491.45)	16.831.90 (1.971.90)	16.751.90 (1.971.90)	16.751.90 (1.971.90)	16.751.90 (1.971.90)
No. of reflections	41817 (1946)	35631 (2614)	13390 (823)	14575 (891)	14554 (885)	14580 (886)
No. of unique reflections	12710 (752)	14336 (1069)	6358 (642)	6342 (636)	6340 634)	6340 (634)
Multiplicity	3.29 (2.59)	2.49 (2.45)	2.1 (1.3)	2.3 (1.4)	2.3 (1.4)	2.3 (1.4)
Completeness (%)	97.6 (79.7)	98.1 (100.0)	98.2 (99.1)	99.6 (99.1)	99.6 (99.1)	99.6 (99.1)
*R*_merge_ ‡ (%)	3.9 (13.7)	5.0 (45.6)	3.0 (16.1)	4.0 (26.7)	4.0 (24.1)	3.9 (25.6)
*I*/**(*I*)	19.21 (10.45)	14.00 (2.52)	25.89 (3.73)	19.38 (2.39)	18.40 (2.48)	19.24 (2.57)
Space group	*R*3	*R*3	*R*3	*R*3	*R*3	*R*3
No. of molecules per asymmetric unit	2	2	2	2	2	2
Unit-cell parameters						
*a* ()	80.85	81.36	81.13	80.69	80.64	80.65
*c* ()	33.36	33.40	33.47	33.33	33.32	33.31
Average mosaicity ()	0.521	0.286	1.12	1.16	1.16	1.16
Refinement statistics						
Resolution cutoff ()	1.50	1.45	1.90	1.90	1.90	1.90
No. of atoms in the model						
Total non-H atoms	906	922	860	865	870	861
Total H atoms	792	793	0	0	0	0
Total protein atoms	1616	1615	790	811	811	803
Water molecules	80	98	68	52	57	56
Total *M* ^2+^ ions	2	2	2	2	2	2
*B* factors§ (^2^)						
Overall	29	24	30	27	28	22
Main chain	23	19	27	23	24	19
Side chains and water molecules	32	26	32	30	31	25
*M* ^2+^ ions	14	15	19	18	21	16
R.m.s. deviation from ideal						
Bond lengths ()	0.016	0.014	0.008	0.007	0.008	0.007
Angles ()	1.484	1.469	1.024	1.008	1.047	1.034
Ramachandran plot¶ (%)						
Residues in core regions	98.9	98.9	97.7	97.7	97.7	98.9
Outliers	1.1	1.1	2.3	2.3	2.3	1.1
*R* factors††						
*R*	0.1819	0.1686	0.1663	0.1655	0.1654	0.1686
*R*_free_	0.2243	0.2044	0.2349	0.2195	0.2016	0.2199

† Beamline I911-2, MAX II, MAXIV Laboratory.

‡ *R*_merge_ is defined as 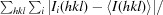 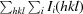, where *I*(*hkl*) is the mean intensity of a set of equivalent reflections.

§ The *B*-factor analysis was performed using *BAVERAGE* included in *CCP*4 (Winn *et al.*, 2011 ▶).

¶ The definition of the Ramachandran plot regions is according to Kleywegt Jones (1996 ▶).

†† *R* and *R*_free_ = 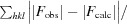 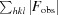, where *F*_obs_ and *F*_calc_ are the observed and calculated structure-factor amplitudes, respectively. *R*_free_ was calculated with a random 5% subset of all reflections that was excluded from the refinement.

**Table 2:** XAS data-collection specifications

	EXAFS		XANES		
	Ni insulin	Cu insulin	Cu insulin		
*K* edge (eV)	8333	8979	8979	8979	8979
Scan range (eV)	81839333	88299829	88599099	88599099	88599099
Scan time per spectrum (min)	55	35	10	10	10
Saccharose protection	No	Yes	No	Yes	Yes
Temperature (K)	100	20	100	100	20
No. of scans	3	3	12	8	29
Dose per scan (MGy)	0.50	0.30	0.09	0.09	0.09

**Table:** Distances are compared with crystallographic values.

	XRD	Restrained EXAFS	
	*R* † ()	*R* ()	2^2^ (^2^)
N^2^ (HisB10)	2.07	2.077 (6)	0.004 (1)
C^1^	3.02	3.09 (3)‡	0.010 (3)
C^2^	3.09	3.03 (3)‡	0.010 (3)
N^1^	4.15	4.22 (2)‡	0.009 (4)
C	4.21	4.21 (3)‡	0.009 (4)
C	5.65	5.56 (5)‡	0.009 (4)
O^w1^ (off *C*_3_ axis)	2.18	2.10 (1)	0.016 (3)
O^w2^ (on *C*_3_ axis)	3.40	2.84 (3)	0.011 (6)

**Table:** EXAFS refinement statistics.

*E*_f_ (eV)	0.40
Fit index _v_^2^	0.48
*R*_exafs_ (%)	15.14
*R*_dist_ (%)	1.12
*R*_total_ (%)	16.26
*N*_p_	19
*k* range (^1^)	2.814.0
*w*_dist_	0.5

† Average distances of both Ni sites in the crystal structure.

‡ The angle (polar coordinates) was refined in order to allow some movement of the atoms in the restrained imidazole ring.

**Table 4:** Optimized structural parameters and statistics for constrained EXAFS refinement of four different coordination geometries of Cu insulin Values marked with an asterisk have been restrained to be equal to a previous value owing to the symmetry of the model.

	Trigonal symmetric hexacoordinated	Tetragonally elongated hexacoordinated	Tetragonally distorted pentacoordinated	Pseudo-tetrahedral tetracoordinated
CuN^2^ ()	1.99 (1)	1.98 (1)	2.04 (1)	1.99 (1)
CuN^2^ ()	1.99 (1)*	1.98 (1)*	2.04 (1)*	1.99 (1)*
CuN^2^ ()	1.99 (1)*	2.02 (1)	1.98 (1)	1.99 (1)*
CuO^w^ ()	2.15 (2)	2.17 (2)	2.23 (3)	2.11 (3)
CuO^w^ ()	2.15 (2)*	2.17 (2)*	2.23 (3)*	
CuO^w^ ()	2.15 (2)*	2.37 (2)		
*N*_p_	8	10	9	8
Fit index **_v_^2^	7.00	2.11	2.68	2.67
*R*_exafs_ (%)	25.66	14.38	15.48	15.60

**Table 5:** Tetragonally distorted hexacoordinated Cu coordination distances and DebyeWaller factors as refined from constrained EXAFS of copper insulin compared with crystallographic values The constrained EXAFS does not distinguish between sites I and II.

	XRD	Constrained EXAFS†		
	*R* ‡ ()	*R*_1_ ()	*R*_2_ ()	2^2^ (^2^)
N^2^ (HisB10)	2.10	1.98 (1)[Table-fn tfn10]	2.02 (1)[Table-fn tfn10]	0.002 (1)
C^1^	3.01	3.08	2.98	0.012 (5)
C^2^	3.15	2.97	3.16	0.012 (5)
N^1^	4.17	4.16	4.13	0.016 (3)
C	4.25	4.11	4.22	0.016 (3)
C	5.68	5.53	5.67	0.016 (3)
O^w^	2.25 (site I, 6-coordinated)	2.17 (2)	2.37 (2)	0.011 (5)
O^w^	2.67 (site II, 4-coordinated)			

† In the tetragonally distorted hexacoordinated geometry the distances to ligands in the tetragonal plane (*R*_1_) are refined separately from the axial ligands (*R*_2_), whereas the DebyeWaller factors are grouped. The occupation numbers *N* of histidine ligands and water ligands are 2 in the plane and 1 on the axis.

‡ Average distances from Cu to histidine and water atoms of both Cu sites in the crystal structure.

§ The rotation angle of the histidine unit around an axis orthogonal to the imidazole plane passing through the N^2^ atom was included in the refinement.
